# Arterial Levels of Oxygen Stimulate Intimal Hyperplasia in Human Saphenous Veins via a ROS-Dependent Mechanism

**DOI:** 10.1371/journal.pone.0120301

**Published:** 2015-03-23

**Authors:** Binata Joddar, Michael S. Firstenberg, Rashmeet K. Reen, Saradhadevi Varadharaj, Mahmood Khan, Rachel C. Childers, Jay L. Zweier, Keith J. Gooch

**Affiliations:** 1 Department of Biomedical Engineering at The Ohio State University, Columbus, OH 43210, United States of America; 2 Davis Heart & Lung Research Institute at The Ohio State University, Columbus, OH 43210, United States of America; 3 Division of Cardiothoracic Surgery at The Ohio State University, Columbus, OH 43210, United States of America; 4 Department of Emergency Medicine at The Ohio State University, Columbus, OH 43210, United States of America; University of Giessen Lung Center, GERMANY

## Abstract

Saphenous veins used as arterial grafts are exposed to arterial levels of oxygen partial pressure (pO_2_), which are much greater than what they experience in their native environment. The object of this study is to determine the impact of exposing human saphenous veins to arterial pO_2_. Saphenous veins and left internal mammary arteries from consenting patients undergoing coronary artery bypass grafting were cultured ex vivo for 2 weeks in the presence of arterial or venous pO_2_ using an established organ culture model. Saphenous veins cultured with arterial pO_2_ developed intimal hyperplasia as evidenced by 2.8-fold greater intimal area and 5.8-fold increase in cell proliferation compared to those freshly isolated. Saphenous veins cultured at venous pO_2_ or internal mammary arteries cultured at arterial pO_2_ did not develop intimal hyperplasia. Intimal hyperplasia was accompanied by two markers of elevated reactive oxygen species (ROS): increased dihydroethidium associated fluorescence (4-fold, p<0.05) and increased levels of the lipid peroxidation product, 4-hydroxynonenal (10-fold, p<0.05). A functional role of the increased ROS saphenous veins exposed to arterial pO_2_ is suggested by the observation that chronic exposure to tiron, a ROS scavenger, during the two-week culture period, blocked intimal hyperplasia. Electron paramagnetic resonance based oximetry revealed that the pO_2_ in the wall of the vessel tracked that of the atmosphere with a ~30 mmHg offset, thus the cells in the vessel wall were directly exposed to variations in pO_2_. Monolayer cultures of smooth muscle cells isolated from saphenous veins exhibited increased proliferation when exposed to arterial pO_2_ relative to those cultured at venous pO_2_. This increased proliferation was blocked by tiron. Taken together, these data suggest that exposure of human SV to arterial pO_2_ stimulates IH via a ROS-dependent pathway.

## Introduction

Saphenous veins (SV), which are widely used as coronary artery bypass grafts (CABG), develop significant intimal hyperplasia (IH) characterized by increased intimal thickening and cellular proliferation as early as 2 weeks following grafting into the arterial circulation [1]. This IH is believed to predispose the vein grafts to atherosclerosis [1]. The observations that SV rarely, if ever, develop significant atherosclerosis in their native venous environment and the rapidity that they develop IH following grafting suggests that aspects of the arterial environment stimulate IH.

While vein graft failure is ultimately an in vivo phenomenon, a number of studies employing the ex vivo culture of intact veins have been performed to explore factors influencing the development of IH. A primary benefit of these ex vivo models is that they afford much better control and monitoring of the mechanical and chemical environments than possible with vessels in vivo while allowing the study of whole-vessel behavior not captured in cell culture. Excised human and porcine veins cultured ex vivo under static mechanical conditions (i.e., no/minimal flow and pressure) develop significant IH and have been used to study the effects of pre-existing IH [2], surgical preparation [3], and specific biochemical factors, such as basic fibroblast growth factor (bFGF) [4] and endothelin1 (ET-1) [5] on the extent of IH developed during culture. To explore the role of the mechanical environment on IH, SV have been cultured in perfusion systems that subject the SV to various levels of pressure, flow, and pulsatility [6,7,8]. In these perfusion systems, the extent of medial hypertrophy is directly related to transmural pressure while IH is inversely related to the magnitude of flow induced shear stress [6,7,8]. The data from these perfusion studies suggest that exposure to arterial hemodynamics is not the primary stimulus of vein graft IH since veins develop significant IH under static conditions and under venous levels of flow. Instead, the extent of IH decreases with increasing mechanical loading with the least IH occurring under mechanical conditions that mimic the arterial circulation [6,7,9].

We recently reported that SV excised from young, healthy pigs and perfused ex vivo with arterial pO_2_, but not those perfused with lower pO_2_, developed IH as evidence by increased cellular proliferation and intimal thickness relative to freshly isolated SV [10]. The pO_2_-induced IH occurred in the absence of exposure to an arterial mechanical environment with IH occurring both in porcine SV cultured under venous and static (no flow) mechanical conditions. Here we use human SV and internal mammary artery (IMA) segments from patients receiving CABG to determine the effects of arterial pO_2_ on IH in clinically relevant vessels. Given previous work implicating ROS in the development of IH in SV grafted in vivo [11] and cultured ex vivo [12], specific attention was given to the levels and role of ROS in this system.

## Methods

### Human vessel harvest and preparation

Use of human tissue was approved by the Biomedical Science Institutional Review Board at the Ohio State University. SV segments were collected from 100 patients who had provided written consent. From this group of 100 patients, however, there was an adequate length of IMA not needed for bypassing to allow harvest of IMA segments from only 4 patients. All veins were harvested with standard endovascular techniques or with limited skin incisions used only for identifying large branches or for harvest of single short segments. After construction of indicated bypass grafts in patients undergoing CABG, the residual segments of SV that would otherwise be discarded were obtained. Vessels from patients with varicose veins or communicable diseases were excluded. After harvesting, all SVs were washed in heparinized saline and flushed to identify small side branches. Attention was given not to distend any vein. After standard median sternotomy, the left internal mammary (IMA) was harvested as a pedicled graft using low energy electrocautery. All branched IMAs, depending on size, were either clipped or cauterized. Following full anticoagulation, the distal IMA was clipped and sharply divided. Prior to anastomosis to the left anterior descending artery, any extra length, when clinically appropriate, was sharply divided. All vessels (SV and IMA) were transported to the laboratory in a gas-impermeable chamber containing ~100 cc of culture medium pre-equilibrated with the desired gas mixture and pre-warmed to 37ºC.

### Ex vivo organ culture and oxygen environments

The time from initial vessel harvest to culture set-up never exceeded 3 hours. Vessels were cultured in low-glucose DMEM supplemented with 10% FBS, 100 μg/ml penicillin, 100 μg/ml streptomycin, 0.25 μg/ml amphotericin B, and 25 mM HEPES as described [12]. SV segments were cultured with an atmospheric pO_2_ of 40 mm Hg (~venous pO_2_), 95 mm Hg (~arterial pO_2_), or at 140 mm Hg (typical cell culture atmosphere of 5% CO_2_ balance air) for 14 days. Every two days, culture medium was changed to fresh medium pre-equilibrated to desired pO_2_. For select vessels, medium was supplemented with 100 μM tiron (Sigma, St. Louis, MO).

### Histology

Histological sections were stained with modified Verhoeff Van Gieson elastin stain kit (Sigma) and counter stained with hematoxylin. Elastin staining was used to estimate the intimal and medial areas of vessels, which were delineated by the external (EEL) and internal elastic lamina (IEL) and quantified using Image J (NIH). Intimal area was determined by quantifying the tissue area above the IEL. Proliferating cells in SV were identified with monoclonal mouse PC 10 antibody recognizing proliferating cell nuclear antigen (PCNA, DAKO). Immunostaining for von Willebrand factor (vWF, Chemicon) was used to detect the presence of endothelium in SV. The extent of muscularization was examined by immunostaining with an anti-alpha smooth muscle actin (αSMA) antibody (Sigma) visualized with DAB substrate and counter stained with Shandon Hematoxylin (Thermo Scientific). PCNA and vWF stained sections were counterstained with DAPI (Vector). Mitotic index, the percentage of proliferating cells, is calculated by dividing the number of PCNA positive nuclei divided by the total number of DAPI labeled nuclei.

### ROS detection and quantification

Levels of ROS in SV were assessed using conversion of non-fluorescent dihydroethidium (DHE) to fluorescent ethidium bromide [13]. Briefly vein sections from freshly isolated and 14-day old cultures were frozen in optimum cutting temperature compound media (Tissue-Tek; Sakura Finetechnical, Tokyo, Japan). Cryo-sections (10 μm thick), were prepared and incubated with dihydroethidine (DHE; 10 μM) for 30 min at 37°C under dark conditions and imaged within 5 min. In all cases, the incubation with DHE and subsequent imaging was conducted under the same levels of pO_2_ (~140 mmHg). PEG-SOD (170 IU/ml; Sigma Aldrich, MO) was used to scavenge superoxide in select sections to confirm the role of superoxide in the observed fluorescence. While it is extremely unlikely that cells in cryosections are viable, previous studies by the authors have shown that under these conditions, the magnitude of red fluorescence qualitatively agrees with the ROS levels assessed using HPLC [e.g,[14] or electron paramagnetic resonance [e.g., [15]. Image J was used to determine the pixel intensity histogram, i.e., the number of pixels n_I_ at each intensity I. From these data, the average pixel intensity for an image $\overset{-}{I}$ was calculated as $\bar{I}=\frac{\sum_{I=0}^{255}I*n_{I}}{\sum_{I=0}^{255}n_{I}}.$ The average pixel intensity for images corresponding to various culture conditions were normalized to the $\overset{-}{I}$ of freshly harvested SV.

### Analysis of 4-hydroxynonenal (4-HNE) by immunostaining and western blotting

Immunostaining was performed using 4-HNE polyclonal antibodies (Bethyl Labs, Montgomery, TX). Previously frozen tissue was homogenized and lysed for Western blot analysis using 4-HNE polyclonal antibodies (Axxora, San Diego, CA).

### Electron paramagnetic resonance

The pO_2_ measurements were performed using an EPR spectrometer (Magnettech GmbH; Berlin, Germany) equipped with automatic coupling and tuning controls. Microcrystals of lithium octa-n-butoxy-naphthalocyanine (LiNc-BuO), an oxygen sensing probe, were used for EPR oximetry [16]. LiNc-BuO, crystals with a diameter less than 50 μm, were suspended in PBS at a concentration of 2 mg/ml. 10 μl of this LiNc-BuO suspension was injected into the SV wall using a 24-guage needle. The SV was then cultured ex vivo with the desired pO_2_ for at least 24 hours before subjecting it to EPR measurements. EPR spectra were acquired as single 30-sec duration scans. The instrument settings used were: microwave frequency, 1.2 GHz (L-band), incident microwave power, 4 mW; modulation amplitude, 180 mG, modulation frequency 100 kHz; receiver time constant, 0.2 s. The peak-to-peak width of the EPR spectrum was used to calculate pO_2_ using a standard calibration curve [16,17].

### SMC isolation

SMC were isolated from freshly isolated SV segments using standard explant techniques [18]. SV segments were denuded of their endothelium, stripped of their adventitia, cut into ~ 1 mm squares, and placed on tissue-culture treated plastic and feed with culture medium. Once cells had migrated out of the tissue, the tissue was removed and discarded. Smooth muscle cell phenotype was confirmed by immunocytochemical staining with an anti-smooth muscle actin antibody (Dako). SMC from passages 3–8 were used.

### Statistical analysis

Unless noted, all data are reported as means ± standard deviation. Data from paired study designs were analyzed using Student’s paired t-test and multiple groups compared using Bonferroni’s corrections. Except where noted, for each experiment or condition, n ≥6 with the SV segments coming from 6 or more patients. Using normalized values help ensure that variations in endpoints such as amount of IH reflect real changes in the vessel during culture and not the difference in the starting material due to patient-to-patient variability. *p*<0.05 was considered statistically significant.

## Results

### Ex vivo culture with arterial pO2 stimulates IH in SV but not in IMA

**Fig 1:**
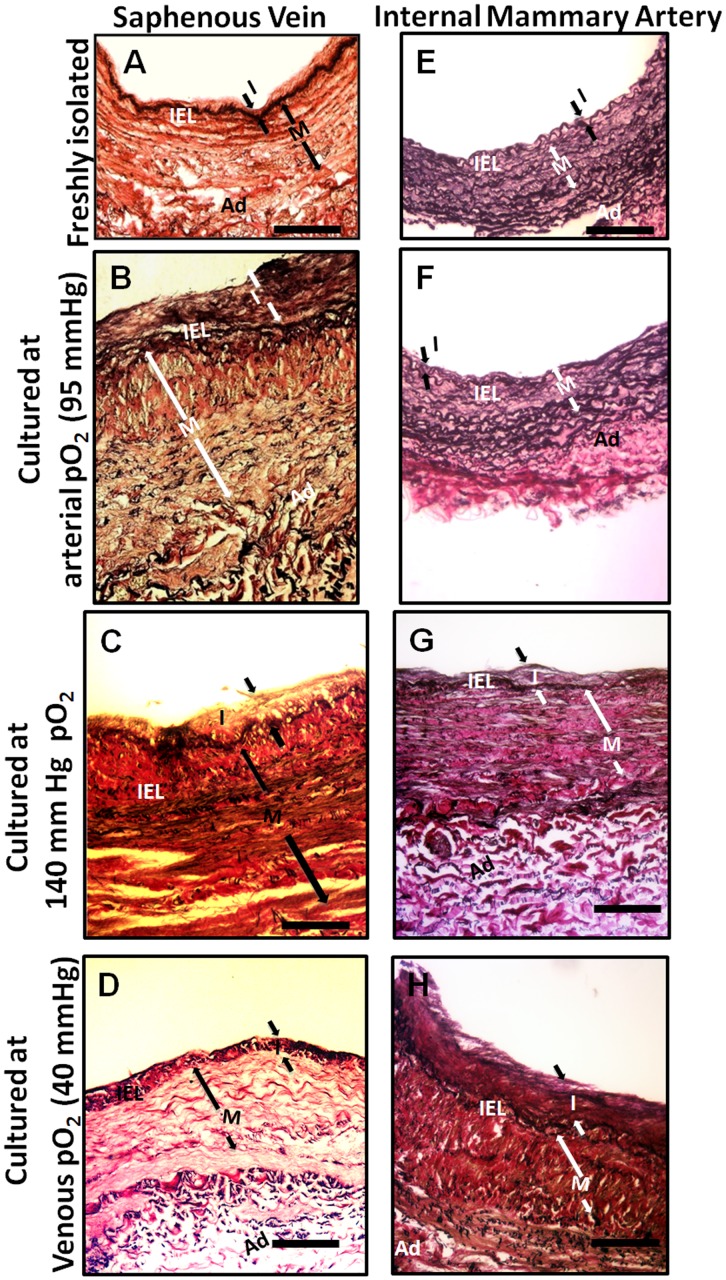
Histology of human SV and IMA freshly isolated or cultured with arterial pO_2_. Human SV (A-D) and IMA (E-H-) stained with Elastin Stain for both freshly isolated vessels (A,E) and vessels cultured at arterial pO_2_ (B,F), typical cell culture pO_2_ (C,G) and venous pO_2_ (D,H). The intima (I), media (M), adventitia (Ad) and inner elastic lamina (IEL) are labeled when visible. Scale bar is 100 μm.

**Fig 2:**
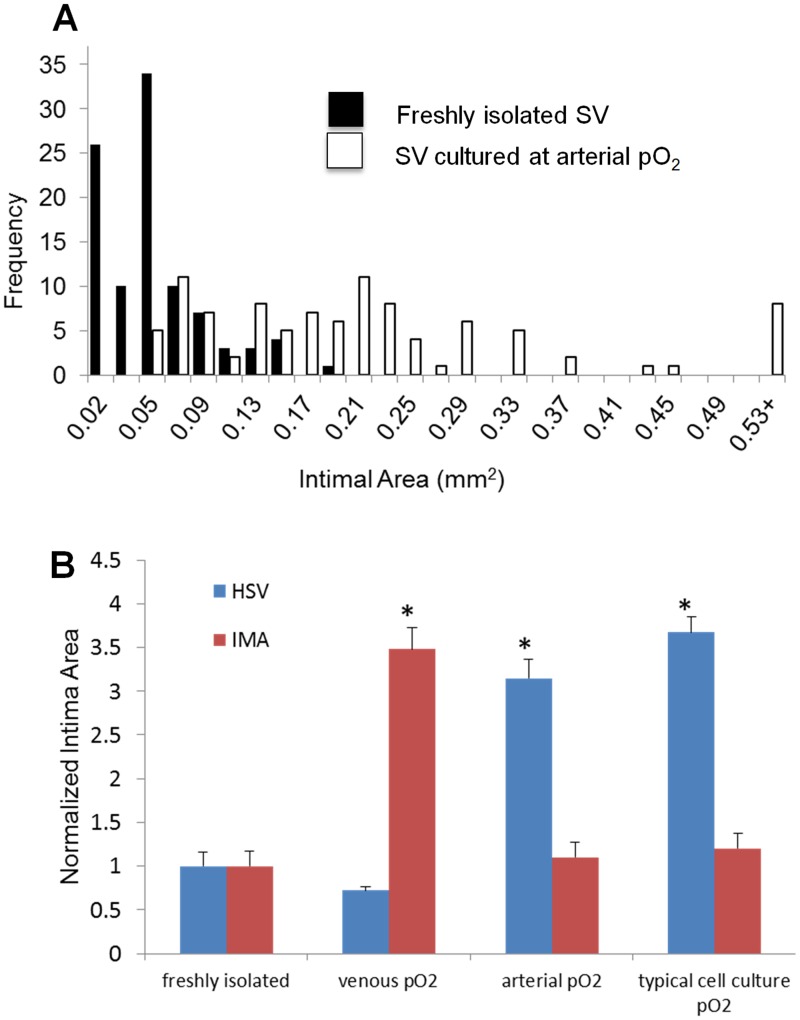
Quantification of intimal area. (A) Histogram of intimal areas for freshly isolated SV and SV cultured at arterial pO_2_. (B) Normalized intimal area of SV (n≥6) and IMA (n = 4) when either freshly harvested or cultured at venous, arterial and typical cell culture pO_2_. * indicates p<0.05 relative to other groups.

Relative to freshly harvested SV, those cultured for 14 days with arterial pO_2_ exhibited an increase in intimal area, a region in the inner portion of the media where the SMC lost their typical circumferential alignment, and exhibited medial thickening (Fig. 1A,B). None of these histological changes were observed in IMA that were cultured using arterial pO_2_ (Fig. 1E,F). The freshly harvested SV segments from 100 patients exhibit a wide range of initial intimal areas but consistently increased their intimal area when cultured with arterial pO_2_ (Fig. 2A). To help insure that differences in intimal area observed in vessels cultured under various conditions are due to differences in the culture conditions and not variations in the initial intimal area, the final intimal area for each vessel was normalized to its initial intimal area. The normalized intimal area for SV cultured at arterial pO_2_ is 3.1 (i.e., 3.1 times that of their initial intimal area) (Fig. 2B). SV cultured under typical cell culture conditions of 5% CO_2_ and a balance of humidified air were exposed to a PO_2_ of 140 mmHg, which is greater than arterial pO_2_. SV cultured under these typical cell culture conditions also exhibited IH (Fig. 1C and 2B). In contrast to the IH seen in SV cultured at arterial or higher pO_2_, neither SV cultured at venous pO_2_ (Fig. 1D and 2B) or IMA cultured at arterial pO_2_ (Fig. 1F and 2B) exhibited IH. While the response of IMA to reduced pO_2_ is not the focus of this study, it is noteworthy that they exhibited intimal thickening when cultured under these conditions (Figs. 1H and 2B).

### Culture with arterial pO2, but not venous pO2, stimulates IH in SV

**Fig 3:**
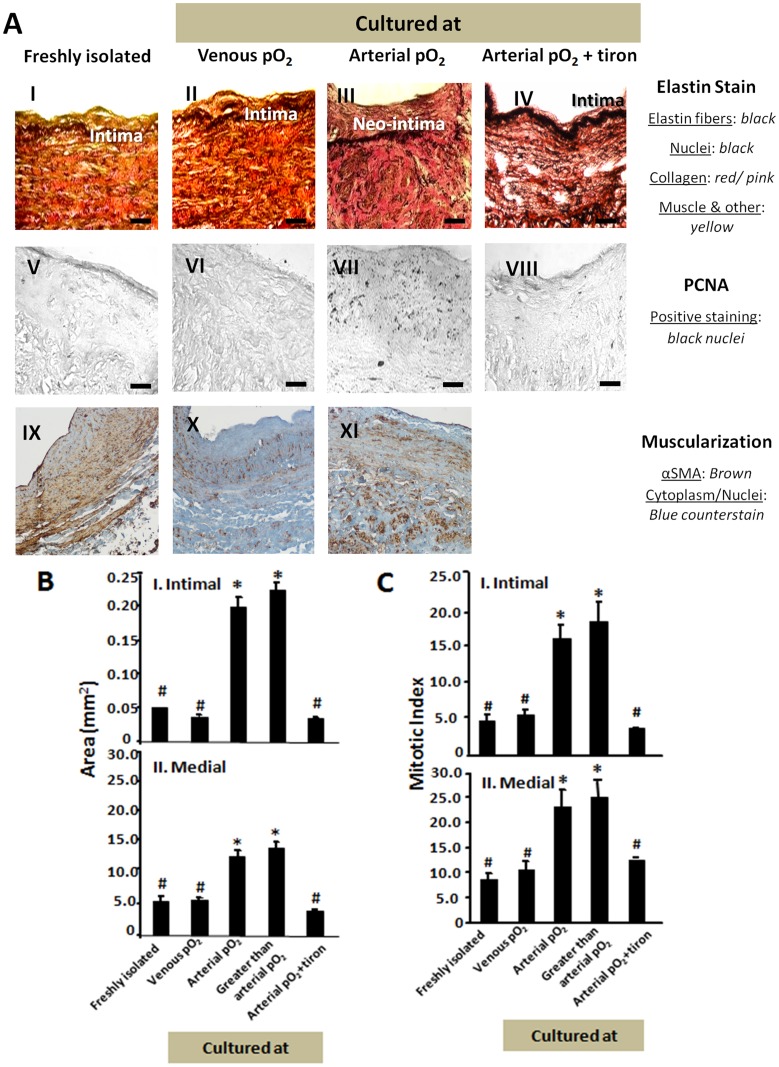
Oxygen-induced remodeling in SV. (A) Markers of hyperplasia and muscularization. Top row shows elastin stained SV freshly isolated (I), and after culture at venous pO_2_ (II), arterial pO_2_ (III), or arterial pO_2_ with tiron added (IV). Neo-intima formation is observed when cultured at arterial pO_2_ without tiron. Elastin fibers and nuclei are stained in black, collagen in red/pink and muscle and other components in yellow. The middle row shows PCNA staining for corresponding conditions (V-VIII). PCNA positive staining shows black nuclei, indicating proliferating cells. Scale bar is 50 μm. The bottom row show αSMA staining (IX-XI). (B) Quantification of intimal (I) and medial (II) areas in mm^2^ is given for SV cultured at 40, 95, or 140 mmHg pO_2_ as well as arterial pO_2_ with tiron. Arterial pO_2_ is synonymous with 95 mmHg. * indicates p<0.05 relative to other groups marked with #. There were no intragroup differences among subgroups marked with # or *. (C) Quantification of intimal (I) and medial (II) proliferation rates in terms of mitotic index is given for SV cultured at 40, 95, or 140 mmHg pO_2_ as well as arterial pO_2_ with tiron. * indicates p<0.05 relative to other groups marked with #. There were no intragroup differences among subgroups marked with # or *.

SV cultured with venous pO_2_ showed no thickening of intimal and medial layers as compared to freshly isolated SV (Fig. 3A- I, II; 3B). In contrast, SV cultured at arterial pO_2_ (95 mmHg, Fig. 3A-III) and at typical cell culture pO_2_ (140 mmHg) had 3.2- and 3.6-fold greater intimal area (Fig. 3B-I) and 2.4- and 2.5-fold greater medial area (Fig. 3B-II) than freshly isolated SV. Since previous studies have implicated an increase in ROS in the IH observed in SV both in vivo [19,20] and ex vivo [12], tiron, a ROS scavenger, was added to SV throughout the 2-week culture period to explore the role of ROS in the observed pO_2_-induced IH. Culturing with tiron, prevented the pO_2_-induced increase in intimal area (Fig. 3A-IV; 3B-I) and medial areas (Fig. 3A-IV; 3B-II). SV cultured at venous pO_2_ exhibited no increase in their mitotic index in the intima or media relative to freshly isolated SV (Fig. 3A-V, VI and 3C). Culture at arterial pO_2_ and typical cell culture pO_2_ caused a 4- and 4.5-fold increase in intimal mitotic index (Fig. 3C-I) and 3-and 3.3-fold increase in medial mitotic index (Fig. 3C-II) compared to freshly isolated SV. Addition of tiron_,_ during culture blocked the pO_2_-induced increases in the mitotic index (Fig. 3C-I, II).

As previously reported by Haefliger and coworkers, [21], the media of freshly isolated SVs positively immunostained for αSMA (Fig. 3A IX). SVs cultured at either venous or arterial pO_2_ also stained for αSMA and there was no consistent difference in the extent of staining in the media between freshly isolated or cultured SV (Fig. 3A IX-XI).

**Fig 4:**
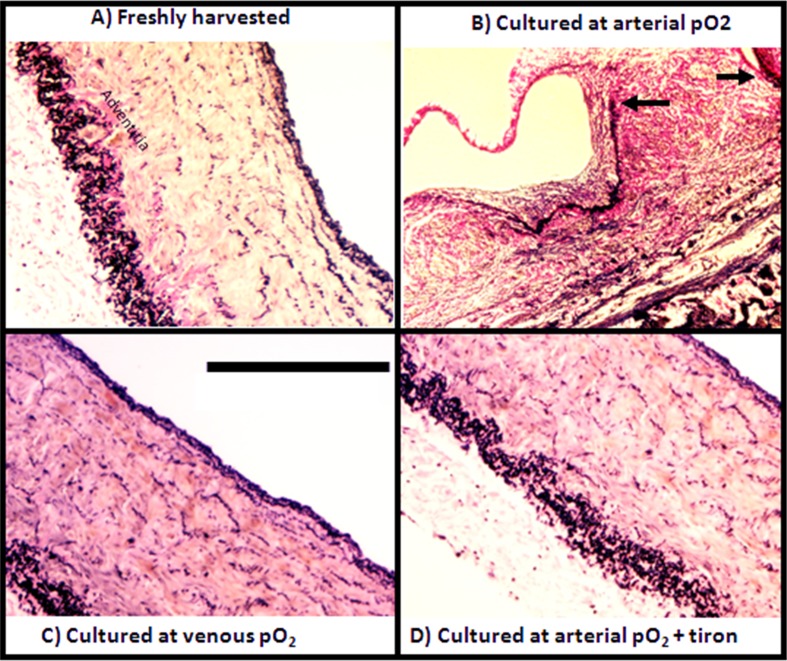
Elastin-stained sections from freshly harvested SV and SV cultured for 14 days. In Panels A, C, and D the and the IEL, which stain black, are intact and there is no neointimal formation. In Panel B there is rupture in the IEL (breaks in IEL indicated with black arrows) allowing cells from the media to migrate into the intima. Scale bar is 100 μm.

As can be more clearly seen in images that show a wider field of view, the IEL, which stains black, is intact in freshly isolated SV (Fig. 4A) and those cultured with venous pO_2_ (Fig. 4C). In contrast, the IEL is disrupted in SV cultured with arterial pO_2_ (Fig. 4B). This disruption allows what appears to be medial tissue to move through the break in the IEL and into intima. Under all conditions tested, the endothelium appeared intact as assessed by immunostaining for von Willebrand (S1 Fig).

SV cultured using venous pO_2_ showed 4.4±0.5% TUNEL-positive nuclei, which was comparable to freshly isolated SV (4.5±0.5%). SV cultured at arterial pO_2_ showed higher rates of TUNEL staining (7.3±0.3%, p = 0.001 relative to freshly isolated). Culture with tiron did not reduce TUNEL staining in vessels exposed to arterial pO_2_ (6.8±0.2%). To better understand the net effect of the ~4 fold-increase in proliferation and the less than 1-fold increase in apoptosis measured at the end of the two week of culture with arterial pO_2_, cell nuclei density in histological sections was assessed using DAPI staining (S1 Fig). Freshly isolated SV and SV cultured with venous pO_2_ had similar cell densities but culture with arterial pO2 increased cell density by 70% (S1 Fig.). Addition of tiron during culture with arterial pO_2_ maintained the cell density at that of freshly isolated SV (S1B Fig.). Relative to freshly isolated vessels, total cell number calculated as the product of cell density and tissue area, increased 4-fold in vessels cultured at arterial pO_2_ suggesting that the increase in proliferation has a stronger impact on cell number than the increase in apoptosis.

### pO2 within the SV wall varies linearly with the pO2 at which the SV is cultured

**Fig 5:**
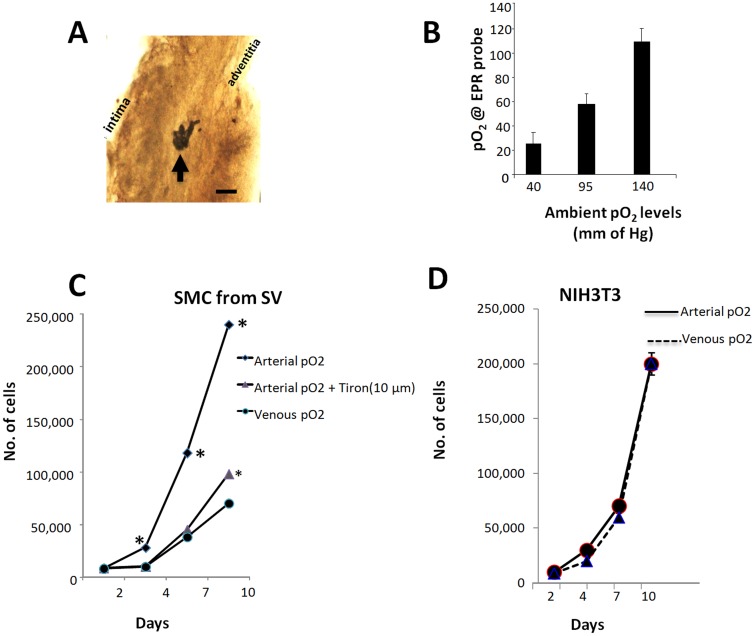
Oxygen levels within the wall of SV cultured at various levels of pO_2_ and the impact of pO_2_ on the proliferation of cultured SMC. (A) Arrow indicates location of the black EPR probe within SV cultured ex vivo for internal pO_2_ measurements. Intima and adventitia are labeled to show vessel orientation. Scale bar is 50 μm. (B) Quantification of oxygen levels within intact SV as measured by EPR probe. SV were in ex vivo culture at pO_2_ of either 40 (venous), 95 (arterial) or 140 mmHg (typical cell culture atmosphere). Cell number as a function of time during culture for SMC isolated from HSV (C) and 3T3 fibroblasts (D). Isolated SMC were cultured at venous pO_2_ or at arterial pO_2_, with and without tiron. NIH3T3 fibroblasts were cultured at venous or arterial pO_2_. *indicates statistically different compared to other groups at corresponding time point. Standard deviations bars are not visible when their size is small compared to the size of the symbols indicating means.

Since cell proliferation was elevated throughout the thickness of the wall of SV cultured at arterial pO_2_, we speculated that exposure of SV to increased pO_2_ might increase the pO_2_ within the vessel wall, which might directly influence SMC proliferation. Oxygen-sensitive LiNc-BuO crystals were injected into the middle portion of the wall of SV wall, which was then cultured at venous, arterial, or above arterial pO_2_. After 4 days, serial histological sections around the injection point were prepared. The dark LiNc-BuO crystals were clearly visible in the middle portion of the SV wall with tissue around the crystals intact; no evidence of the needle tract was present (Fig. 5A). SV segments with implant LiNc-BuO crystals that had been cultured at various pO_2_ levels were then used for EPR to detect the pO_2_ levels at the LiNc-BuO crystals. The levels pO_2_ deep within the vessel wall increased linearly with the pO_2_ levels in the exterior culture medium (Fig. 5B).

### SMC from SV show sensitivity to varying pO2 in the culture

To investigate whether the elevated pO_2_ within the vessel wall could potentially act directly on SMC, the effect of pO_2_ on the proliferation of cultured SMC was investigated. When the SV pieces were cultured with venous pO_2_, no SMC were observed to migrate out from the tissue even after one month. SMC retained their ability to migrate from the tissue, however, since if pO_2_ was changed from venous to higher levels two weeks into culture; SMC subsequently migrated out from the tissue. Since we were interested in studying how SMC proliferation responds to an abrupt increase to arterial pO_2_ (opposed to the transition from arterial to venous pO_2_), we first preconditioned the cells that had explanted at higher pO_2_ by culturing them under venous pO_2_ for at least 10 days. During this time, the cells continued to proliferate and were passaged when they became confluent.

These cultures exhibited hill-and-valley morphology typical of SMC (data not shown) with 95±3% of the cells staining positive for **αSMA**. SMC were then seeded at 10,000 cells per well in a 24-well plate and cultured at venous or arterial pO_2_. After 10 days, there were approximately 3-fold more cells in cultures maintained at arterial pO_2_ than those cultured at venous pO_2_ (Fig. 5C). Addition of tiron to cultures maintained at arterial pO_2_ reduced SMC proliferation to that of those maintained with venous pO_2_ (Fig. 5C). In contrast to human SV SMC, NIH3T3 fibroblast proliferation was not influenced by pO_2_ (Fig. 5D). The results with fibroblasts suggests that greater proliferation of cells exposed to arterial pO_2_ relative to those exposed to venous pO_2_ is not a universal response.

### Culture with arterial pO2, but not venous pO2, increases ROS in SV

**Fig 6:**
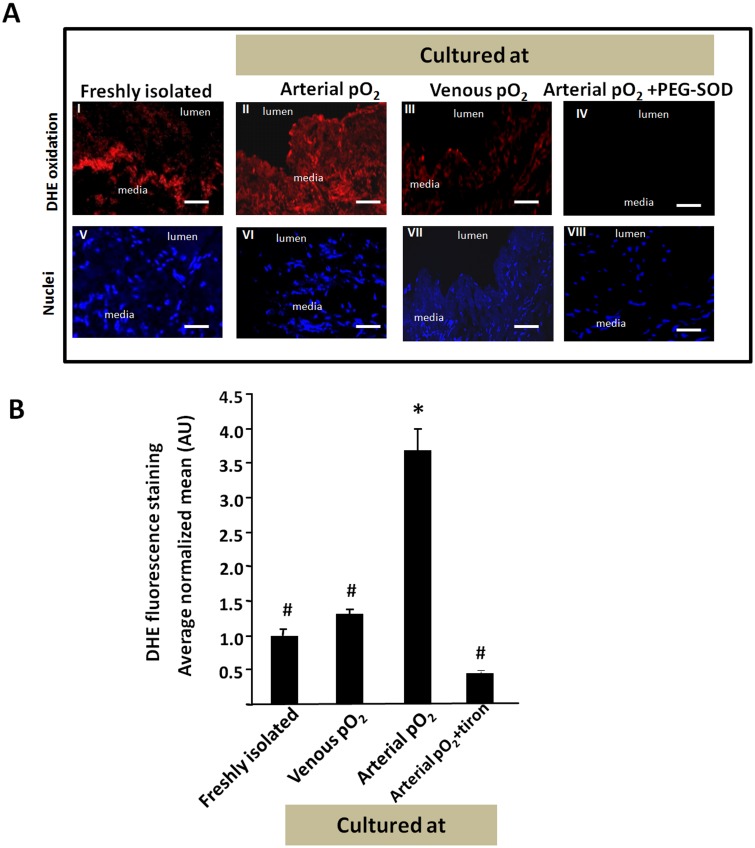
ROS levels in freshly isolated and cultured SV as assessed by DHE staining. (A) DHE and DAPI staining of freshly isolated SV and SV cultured at arterial or venous pO_2_. Scale bar is 100 μm. (B) Average normalized mean of DHE fluorescence intensity (n = 3). * indicates p<0.05 relative to other groups marked with #. There were no intragroup differences among subgroups marked with #.

Since chronic exposure to tiron during culture blocked IH, we speculated that the pO_2_ level at which the vessel was cultured at might influence ROS levels. Consecutive cryosections were stained with either DHE to assess the levels of ROS or DAPI to determine the location of nuclei. SV cultured with arterial pO_2_ exhibit more intense red DHE fluorescence covering a larger fraction of the vessel wall than freshly isolated SV or SV cultured with venous pO_2_ (Fig. 6). The red DHE fluorescence was blocked by pre-treatment with PEG-SOD, indicating its dependence on superoxide (Fig. 6). We have previously reported that relative to freshly isolated vessels, human SV cultured ex vivo at arterial pO_2_ does not decrease the activity of catalase or superoxide dismutase [12] suggesting that the increased red DHE fluorescence was not due to decreased decomposition superoxide or hydrogen peroxide.

### Arterial pO2 increases lipid peroxidation (4-HNE) levels in cultured SV relative to freshly isolated SV or those cultured with venous pO2

**Fig 7:**
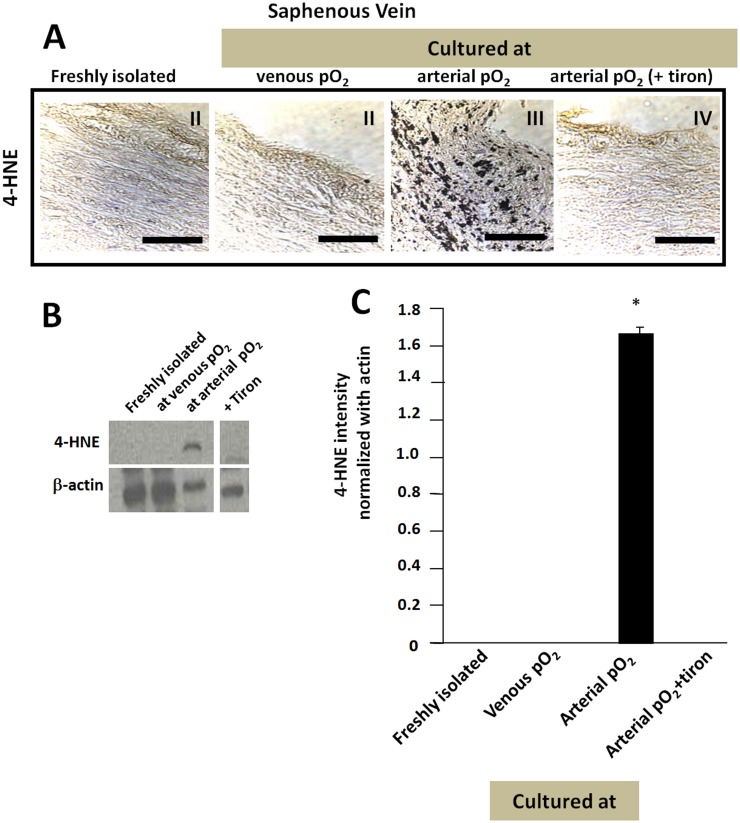
Distribution and levels of 4-HNE adducts in freshly isolated and cultured SV. (A) SV immunostained for 4-HNE adducts. Positive staining is shown in dark brownish black and detects levels of lipid peroxidation due to ROS. Conditions shown are freshly isolated (I), and after being cultured at venous pO_2_ (II) or arterial pO_2_ without (III) and with tiron (VI). Scale bar is 100 μm. (B) Western blot for 4-HNE adducts. Gels contained samples not related to the present study and these lanes are not shown. Noncontiguous gel lanes are demarcated by a vertical white line. (C) Quantification of 4-HNE normalized to actin amount. *p<0.05 relative to group marked with #.

In an effort to collaborate the results from the DHE staining that suggested increased levels of ROS in SV cultured with arterial pO2, lipid peroxidation products, specifically 4-HNE, were assessed using immunohistochemistry and western blots for 4-HNE adducts. SV cultured at arterial pO_2_ showed greater intensity of 4-HNE adduct immunostaining as compared to freshly isolated SV or SV cultured at venous pO_2_ (Fig. 7A). In western blots, 4-HNE adducts were detected only at a single band size of ~48 kDa (Fig. 7B) with the intensity of this band (Fig. 7C) following the same trend seen in immunostaining. Addition of tiron reduced 4-HNE adduct staining to basal levels in both histological sections and western blots (Fig. 7). Since arterial pO_2_ does not decrease the activity of catalase or superoxide dismutase in SV [12], the increased levels of 4-HNE likely indicate increased levels of ROS production.

## Discussion

The IH we report here with SV cultured at arterial (90 mm Hg) and under standard cell culture atmosphere of 5% CO_2_ balance humidified air (140 mm Hg) is similar to that reported by others who have cultured SV under standard cell culture atmosphere [5,22,23]. We believe we are the first to demonstrate that the IH in this widely-used ex vivo model system of human vein graft disease is the result of exposing the vein to elevated pO_2_. These results with human SV presented here as well as previously published results with porcine SV [10] suggest that exposure to arterial pO_2_ might be a primary stimulus for IH in saphenous and other veins that are abruptly transitioned from venous to arterial pO_2_ in vivo. Abrupt transition to arterial pO_2_ occurs in SV used in CABG and peripheral grafts as well as in cephalic veins following the placement of arteriovenous shunts or access graft for hemodialysis. In each of these cases, the veins exposed to elevated pO_2_ develop IH and are at increased risk of developing atherosclerosis. In addition to IH, exposure of human SV to arterial pO_2_ ex vivo results in medial hypertrophy and hyperplasia, consistent with the arterialization of saphenous veins used in CABG [10]. While this arterialization is typically attributed to exposure to the larger pressures found in the arterial circulation [10]], the results presented here suggest that exposure to arterial pO_2_ may also play an important role in the arterialization process.

There have been only a few in vivo studies attempting to elucidate the role of pO_2_ in the development of IH in various vessels and, to our knowledge, none have studied veins. Studies of catheter-induced IH in rabbits chronically exposed to chronic hyperoxia [24] or hypoxic [25] atmospheres are difficult to interpret since both atmospheres have significant impact on blood lipid levels, which themselves are major modulators of IH. When intermittent hyperoxia is induced, it has a much diminished effect on blood lipids. In one study with cholesterol-fed rabbits, intermittent hyperoxia did not alter catheter-induced IH but it doubled the intima area in regions of the arteries not exposed to the balloon catheter [26]. The observation that hyperoxia can stimulate IH in vivo is consistent with our notion that exposure to increased pO_2_ contributes to IH in SV grafted into the arterial circulation. It is acknowledged, however, that there are important differences between these two cases (e.g., rabbits vs. human, artery vs. vein, intermittent vs. continuous exposure to elevated pO_2_) and further studies are needed.

The roles of oxidative stress and ROS often have been discussed in the context of a number of vascular diseases including hypertension, atherosclerosis, and restenosis [27,28]. Superoxide levels, as assessed by SOD-inhibitable nitro blue tetrazolium reduction, are elevated in porcine vein grafts relative to arterial grafts [11]. Our present study has shown increased DHE fluorescence in SV cultured with arterial pO_2_ relative to freshly isolated SV or SV cultured at venous pO_2_. While DHE is widely used to measure superoxide, it is imperfect. Oxidation of the DHE probe can lead to fluorescence independent of superoxide [29]. The ability of PEG SOD to block the fluorescence suggests, but does not prove, its dependence on superoxide. Thus it is important to corroborate the DHE findings with an alternative marker of elevated levels of ROS. ROS leads to peroxidation of lipids to form various products, including 4-HNE. The culture of SV with arterial pO_2_ resulted in increased lipid peroxidation as indicated by the observed increase in levels of 4-HNE adducts. The fact that culturing the vessels in the presence of ROS scavenger tiron blocked the increase in 4-HNE suggests its dependence of elevated levels of ROS.

In addition to being a widely used marker of oxidative stress, 4-HNE is a highly reactive lipid peroxidation product that forms stable 4-HNE adducts with proteins within the tissue, some of which are biologically active and can stimulate SMC growth via altering redox-sensitive mechanisms and growth factor expression [30]. ROS-mediated pathways can also activate members of the MAPK family to lead to cell proliferation via 4-HNE-independent pathways [31]. Thus there are several mechanisms well established in the literature that could potentially link the observed increase in ROS and proliferation.

To our knowledge, no clinical trials have explored the uses of antioxidants for SV grafts, but clinical trials with the vitamins A and E did not show vascular protective effects for restenosis following angioplasty [32,33]. There are significant limitations when using vitamins A and E as antioxidants. For example, despite their reputation as "antioxidant" vitamins, both can act as a pro-oxidant under many conditions [33,34]. Despite their similarities, atherosclerosis, restenosis, and vein graft disease are distinct and it is inappropriate to conclude that failure of a specific antioxidant to demonstrate a beneficial effect for restenosis or atherosclerosis indicates that other antioxidants would not be effective in vein graft disease. Consistent with this notion, we recently reported that both N-acetylcysteine (NAC) and Protandim, a mixture of phytochemicals that increase expression of endogenous antioxidant enzymes, blocked IH in human SV cultured at arterial pO_2_ [12].

Though not a major focus of this study, the observation that SMC failed to explant from SV when cultured under venous pO_2_ but readily did so at higher oxygen levels raises question regarding the phenotype of SMC harvested by the frequently used explant technique. While it is widely acknowledged that SMC start to dedifferentiate with time in culture [35], our observation suggest that even low passage SMC explanted under a typical cell culture atmosphere of 140 mmHg, might already have an altered phenotype more similar to cells contributing to intimal hyperplasia than cells in the SMC with relatively low proliferation seen in SV exposed venous pO_2_.

Taken together, the in vitro and ex vivo studies present herein support the notion that exposing human SV to arterial levels of oxygen stimulates IH characterized by intimal thickening and increased SMC proliferation, with at least a portion of the effect on SMC proliferation the result of changes in their local pO_2_ levels. While exposure of SV to arterial pO_2_ is an unavoidable consequence of their use in CABG and peripheral revascularization, an improved understanding of the process and underlying mechanisms might give insight into potential pharmacological treatments.

## Supporting Information

S1 Fig von Willebrand Factor staining of endothelium and DAPI staining of nuclei. (A) SV stained with von Willebrand Factor to detect endothelium and counterstained with hematoxylin QS (I-IV). Positive staining for endothelium is brown stain seen along upper boundary of sections and black shows elastic fibers and nuclei. Staining with DAPI (V-VIII) shows bright blue indicating cell nuclei. Staining was done on SV freshly isolated, cultured in venous pO_2_, or cultured in arterial pO_2_ with standard conditions or tiron added. Vessels were imaged with lumen facing upward. Scale bar is 100 μm. (B) Number of nuclei per sq. mm of freshly isolated and cultured SV.
(TIF)
