# Supplementary figures and images for: Arterial Levels of Oxygen Stimulate Intimal Hyperplasia in Human Saphenous Veins via a ROS-Dependent Mechanism
Source: PLoS One. 2015 Mar 23;10(3):e0120301. doi: 10.1371/journal.pone.0120301 (PMC4370681; doi:10.1371/journal.pone.0120301)

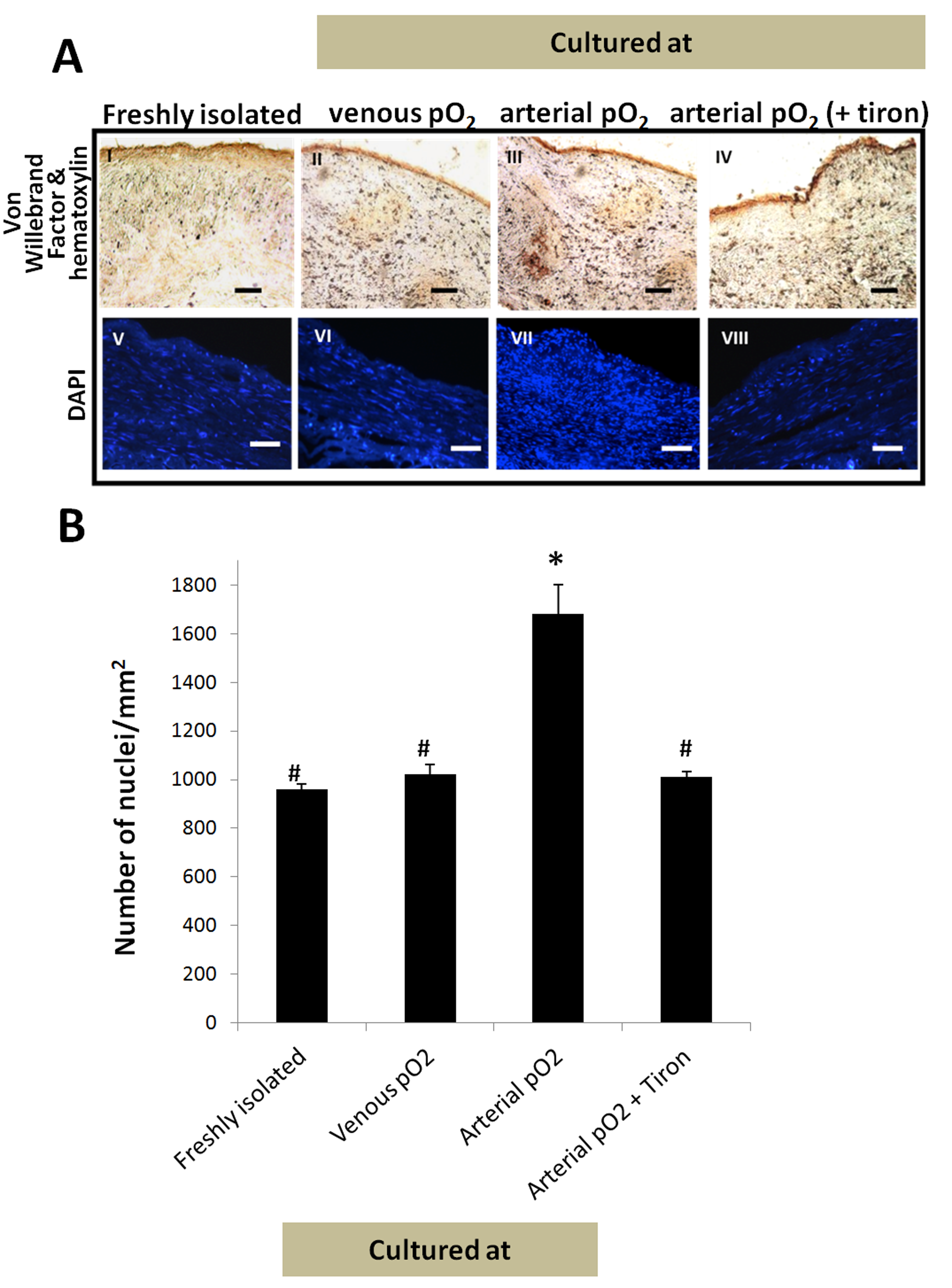

Supplement: S1 Fig — (A) SV stained with von Willebrand Factor to detect endothelium and counterstained with hematoxylin QS (I-IV). Positive staining for endothelium is brown stain seen along upper boundary of sections and black shows elastic fibers and nuclei. Staining with DAPI (V-VIII) shows bright blue indicating cell nuclei. Staining was done on SV freshly isolated, cultured in venous pO2, or cultured in arterial pO2 with standard conditions or tiron added. Vessels were imaged with lumen facing upward. Scale bar is 100 μm. (B) Number of nuclei per sq. mm of freshly isolated and cultured SV. (TIF) [file pone.0120301.s001.tif]
